# Relations between the Arabic BFI-2 and HEXACO-60 scales among Kuwaiti Undergraduates.

**DOI:** 10.1192/j.eurpsy.2024.279

**Published:** 2024-08-27

**Authors:** B. M. Alansari

**Affiliations:** Psychology, Kuwait University, College of Social Sciences, Shuwaikh, Kuwait

## Abstract

**Introduction:**

Many researchers are likely to use the BFI-2 as a measure of the Big Five personality factors. The HEXACO-60 Honesty-Humility factor has no direct counterpart in the Big Five system; however, it should show modest positive correlations with Big Five Agreeableness.

**Objectives:**

The study aimed to examine the BFI-2 in relation to a similar-length version of the HEXACO-60.

**Methods:**

Participants were 1536 undergraduate students (960 women 576 men) at Kuwait university who completed the personality questionnaires. Participants aged 18–23-years-old mean age = 21.26 ± 1.20. The Arabic versions of HEXACO-60 and the BFI-2 instruments were administered in paper-and-pencil format in research laboratories.

**Results:**

Cronbach’s alphas ranged from 0.75 to 0.88 for the BFI-2 Domains and 0.70 to 0.75 for the HEXACO-60 Domains denoting good internal consistency. Regarding cross-inventory correlations, these were high for the two inventories variants of Openness (0.77), Conscientiousness (0.75), and Extraversion (0.71). BFI-2 Agreeableness correlated 0.56 with HEXACO-60 Agreeableness. The HEXACO-60 Honesty-Humility was weakly related to the BFI-2 scales, showing only modest correlation with Agreeableness (0.48). In addition, the BFI-2 Neuroticism correlated 0.53 with HEXACO-60 Emotionality, −0.33 with HEXACO-60 Extraversion, and −0.30 with HEXACO-60 Agreeableness.

**Conclusions:**

The BFI-2 scales captured well the variance of the HEXACO-60 scales apart from Honesty-Humility. In particular, the BFI-2 accounted for about as much variance in the HEXACO Openness, Conscientiousness, Extraversion, and Agreeableness scales as the HEXACO-60 scales accounted for in the BFI-2 scales of the same names. The results confirm the BFI-2 and HEXACO-60 are heavily overlapping.

**Disclosure of Interest:**

None Declared

